# Evidence of housing instability identified by addresses, clinical notes, and diagnostic codes in a real-world population with substance use disorders

**DOI:** 10.1017/cts.2023.626

**Published:** 2023-09-04

**Authors:** Daniel R. Harris, Nicholas Anthony, Dana Quesinberry, Chris Delcher

**Affiliations:** 1 Department of Pharmacy Practice and Science, Institute for Pharmaceutical Outcomes & Policy, College of Pharmacy, University of Kentucky, Lexington, KY, USA; 2 Kentucky Injury Prevention and Research Center, University of Kentucky, Lexington, KY, USA; 3 Department of Health Management and Policy, College of Public Health, University of Kentucky, Lexington, KY, USA

**Keywords:** Social determinants of health, housing instability, natural language processing, geocoding

## Abstract

**Introduction:**

Housing instability is a social determinant of health associated with multiple negative health outcomes including substance use disorders (SUDs). Real-world evidence of housing instability is needed to improve translational research on populations with SUDs.

**Methods:**

We identified evidence of housing instability by leveraging structured diagnosis codes and unstructured clinical data from electronic health records of 20,556 patients from 2017 to 2021. We applied natural language processing with named-entity recognition and pattern matching to unstructured clinical notes with free-text documentation. Additionally, we analyzed semi-structured addresses containing explicit or implicit housing-related labels. We assessed agreement on identification methods by having three experts review of 300 records.

**Results:**

Diagnostic codes only identified 58.5% of the population identifiable as having housing instability, whereas 41.5% are identifiable from addresses only (7.1%), clinical notes only (30.4%), or both (4.0%). Reviewers unanimously agreed on 79.7% of cases reviewed; a Fleiss’ Kappa score of 0.35 suggested fair agreement yet emphasized the difficulty of analyzing patients having ambiguous housing situations. Among those with poisoning episodes related to stimulants or opioids, diagnosis codes were only able to identify 63.9% of those with housing instability.

**Conclusions:**

All three data sources yield valid evidence of housing instability; each has their own inherent practical use and limitations. Translational researchers requiring comprehensive real-world evidence of housing instability should optimize and implement use of structured and unstructured data. Understanding the role of housing instability and temporary housing facilities is salient in populations with SUDs.

## Introduction

Social determinants of health (SDOH), such as living environment and housing stability, heavily influence an individual’s general well-being, and unstable housing itself can have severe negative consequences [1]. Housing deprivation, or homelessness, is the most extreme form of housing instability and can lower life expectancy by 12 years [2]. Approximately 580,000 people experienced homelessness on a single night in 2020 in the USA [3]. The Kentucky Housing Corporation manually counted 3,984 people in 2022 who were unsheltered, living in emergency shelters, or living in some type of transitional housing in Kentucky [4]. Homelessness is associated with significantly higher hospital readmission rates [5], longer hospitalizations [6], disproportionately higher use of emergency medical services and ambulance transports [7], and higher rates of illness and disability [8]. Adults experiencing homelessness have far higher rates of substance use and mental health disorders [9,10]. An in-depth analysis of California’s unhoused population, the largest in the USA, identified that 65% had ever used amphetamines, 56% used amphetamines regularly, and 33% regularly used cocaine [11].

Accurate identification of housing instability in healthcare data is essential for research, as coordination of treatment programs for mental health, substance use disorders (SUDs), and social services for housing results in better health outcomes [12]. In examining populations with stimulants and opioid use disorders, we previously observed significant variation in rates of homelessness using structured data across populations with opioid use disorders (lowest), stimulant use disorders, and concurrent stimulant/opioid use disorders (highest) [13]. Those with SUDs and concurrent housing issues are at a higher risk for overdose [14].

Substantial variation exists in how SDOHs, such as housing instability, are documented within electronic health records [15]. There is no consensus definition of homelessness, no best practices for documenting homelessness, and the low usage of housing-related codes within the International Classification of Disease, Clinical Modification (ICD-10-CM) vocabulary for medical diagnoses (diagnostic billing codes) indicate a general need to improve documentation of housing instability [16]. In a previous study on the impact of SDOH on overdose [13], we found that diagnostic billing codes for housing instability were underutilized within our electronic health record after observing cases of patients experiencing homelessness using residential address data or clinical notes that were not diagnostically coded; this limitation required us to further develop comprehensive methods of obtaining real-world evidence of housing instability for our population with SUDs. Other studies have shown that address data can be used to determine homelessness [17,18]. A systematic review found homelessness was the third most frequent SDOH category actively researched within clinical text (behind smoking status and substance use status) [19]. Clinical text has shown promise in identifying housing issues when paired with natural language processing and data mining techniques, where both lexical approaches requiring a lexicon of housing-related terms and machine learning methods requiring labeled training performed well [20–22]. A rule-based model was useful in identifying housing issues when using unstructured data from multiple hospital systems [23]. We later describe how we use both lexical methods (for identifying housing instability and locations) and model-based methods for recognizing housing-related concepts. This work measures the concordance of housing instability evidence identified by structured, semi-structured, and unstructured clinical data in real-world patient populations having stimulant use disorders, opioid use disorders, and concurrent stimulant/opioid use disorders.

## Materials and Methods

### Patient Population

We extracted records for adult patients who had an encounter with diagnosis codes related to stimulant and opioid use disorders, including poisoning episodes, from the University of Kentucky HealthCare (UKHC) network, which serves central Kentucky with two hospitals, two emergency departments, multiple outpatient clinics, and regional satellite clinics. UKHC is primarily located in Fayette County, Kentucky; the Kentucky Housing Corporation manually counted 715 individuals experiencing housing issues in Fayette County in 2022, which accounts for 17.95% of the entire state’s population facing housing issues [4]. Another 315 (7.9% of the state) individuals were counted in UKHC’s secondary service area, which collectively accounts for 25.85% of the state’s homeless popuation [4]. Our study included 20,556 patients from January 1, 2017 to May 31, 2021. The stimulant-related group was identified with ICD-10-CM diagnosis codes for cocaine use disorders (F14.*), other stimulant use disorders (F15.*), poisoning by cocaine (T40.5*), and poisoning by psychostimulants (T43.6*). The opioid-related group was identified as those with codes for opioid-related disorders (F11.*) and opioid-related poisoning codes (T40.0*, T40.1*, T40.2*, T40.3*, T40.4*, and T40.6*). This study was approved by the University of Kentucky IRB (#74501).

### Structured and Semi-Structured Data

Our electronic health record (EHR) system contains structured ICD-10-CM codes associated with every patient encounter for billing purposes. For problems related to housing and/or low-income economic circumstances, we use ICD-10-CM code Z59*. Semi-structured address data are captured by the EHR as a collection of free-text fields, which includes two address lines, city, state, and zip code. Manual review of addresses revealed patients with addresses corresponding to local supportive housing shelters, including homeless shelters and residential SUD treatment facilities. To categorize patients as having housing issues, we curated a list of addresses for housing-related resources available to our community [24,25].

### Unstructured Data

We extracted 18,847,299 notes from patient visits (mode = 1, median = 208, and average = 945 notes per patient). We deployed three strategies for identifying housing issues using the notes: mentions by keyword, mentions by shelter name, and concepts extracted using named-entity recognition (NER) with a biomedical model. Keywords and phrases were constructed using common knowledge of housing-related words (e.g., “homeless,” “unhoused,” and “unstable housing”). We reused the same list of community resources in the address analysis to explicitly look for mentions of local shelters by name; for example, we observed phrases such as “discharged to Shelter-X,” “lives at Shelter-X,” and “transported from Shelter-X.” Our third strategy was to extract concepts from the text using NER methods available in scispaCy [26]; we deployed scispaCy’s large scientific model (“en_core_sci_lg”), which was previously trained to recognize biomedical text; the NER pipeline is responsible for tokenizing, tagging, parsing, and ultimately generating important pieces of text as named entities. This strategy avoids the need to curate a lexicon of terms related to housing that are needed by keyword matching techniques. We developed a custom knowledgebase linker to the 2022 US SNOMED-CT vocabulary that processes entities recognized by scispaCy and yields structured, coded terms in SNOMED-CT [27]; we provide our contributions as open-source software [28].

To evaluate our identifications when using the unstructured clinical notes, three adjudicators manually reviewed a sample of notes pulled by keyword matching, shelter-by-name matching, and NER. Three hundred notes were randomly sampled, where 100 had positive keyword matches, 100 had positive NER matches, and 100 had positive matches for shelters by name. We analyzed agreement of the three adjudicators by calculating Fleiss’ Kappa.

## Results

Table 1 gives descriptive characteristics of our populations with SUDs, which we subdivide into patients with distinct or combined stimulant- and opioid-related codes; sex, race, and age were statistically significant using chi-squared tests (*p*-value < 0.001) per SUD type (stimulant, opioid, or both). The entry for the SUD type with the highest percentage of representation per demographic is emphasized in bold in Table 1. We also give demographics for the state of Kentucky in 2021 from the US Centers for Disease Control and Prevention for comparison to the study demographics [29]. Notable shifts in demographics include having more males in the stimulant group compared to the opioid group (59.7% vs. 47.2%) and far more Black patients in the stimulant group compared to the opioid group (19% vs. 5%). These shifts are also important in understanding risk and protective factors surrounding both overdose and housing issues. We analyzed adjusted standardized residuals to examine differences between observed and expected numbers. For the stimulant cohort, there were a larger number of male, Black, or ages 18–24 years than expected, while a smaller number of female, White, or ages 65+ years than expected. For the opioid cohort, there were a larger number of female, White, or ages 65+ years than expected, while a smaller number of male, Black, or ages 18–24 years than expected. More discussion on how social determinants impact overdose can be found in our prior work [13], which motivated this study and our development of methods for identifying comprehensive evidence of housing issues.

**Table 1.** Demographics for populations with substance use disorders in the UK healthcare system, 2017 to 2021

		Overall	Stimulants	Opioids	Co-DX	*P*-Value	KY reference (2021)
**Number of unique patients**		20,556	6,165 (30%)	**9,667 (47%)**	4,724 (23%)		
**Sex**	Male	10,754 (52.3%)	**3,683** **(59.7%)**	4,568 (47.2%)	2,503 (52.9%)	<0.001	49.3%
	Female	9,801 (47.7%)	2,482 (40.3%)	**5,099** **(52.7%)**	2,220 (46.9%)		50.7%
	Other/unknown	1 (<1%)	0 (0%)	0 (0%)	**1** **(<1%)**		0%
**Race**	White	18,132 (88.2%)	4,860 (78.8%)	8,905 (92.1%)	**4,367** **(92.4%)**	<0.001	84.9%
	Black	1,953 (9.5%)	**1,169** **(19%)**	485 (5%)	299 (6.3%)		8.9%
	Other	51 (<1%)	20 (<1%)	**27** **(<1%)**	4 (<1%)		6.1%
	Unknown	420 (2%)	116 (1.8%)	**250** **(2.5%)**	54 (1.1%)		0%
**Age group (years)**	18–24	545 (2.6%)	**284** **(4.6%)**	152 (1.6%)	109 (2.3%)	<0.001	9.2%
	25–34	4,533 (22.1%)	1,324 (21.5%)	1,839 (19%)	**1370** **(29%)**		9.2%
	35–44	5,806 (28.2%)	1,566 (25.4%)	2,549 (26.4%)	**1691** **(35.8%)**		12.3%
	45–54	4,213 (20.5%)	**1,498** **(24.3%)**	1,727 (17.9%)	988 (20.9%)		12.4%
	55–64	3,249 (15.8%)	**1,083** **(17.6%)**	1,694 (17.5%)	472 (10%)		13.3%
	65+	2,210 (10.8%)	410 (6.7%)	**1,706** **(17.6%)**	94 (1.9%)		17.2%
**Stimulant or opioid poisoning**	False	16,129 (78.5%)	**5,803** **(94.1%)**	6,587 (68.1%)	3,739 (79.1%)	<0.001	
	True	4,427 (21.5%)	362 (5.9%)	**3,080** **(31.9%)**	985 (20.9%)		

Table 2 summarizes the results using multiple methods for identifying housing issues. 14,545 patients (70.8%) had no evidence of housing issues. Using any data source, 29.2% (*n* = 6,011) of our population had evidence of housing instability. Fifty-four percent with both stimulant-related and opioid-related codes had evidence of unstable housing, compared to 30.7% for our stimulant-only group and 16.2% for our opioid-only group. There is a significant relationship between data source (diagnosis codes, address, and notes) and SUD diagnosis type (Fisher’s exact test *p*-values < 0.001). This relationship implies that ignoring a data source would disregard important information about patients with particular SUD diagnosis types.

**Table 2.** Housing instability by data source for populations with substance use disorders

		Overall	Stimulants	Opioids	Co-DX	*P*-value
**Total population**		6,011	1,891	1,569	**2,551**	
**Housing diagnosis code**	False	2,496 (41.5%)	808 (42.7%)	782 (49.8%)	**906 (35.5%)**	<0.001
	True	3,515 (58.5%)	1,083 (57.3%)	787 (50.2%)	**1,645 (64.5%)**	
**Housing address**	False	5,030 (83.7%)	1,544 (81.6%)	1,288 (82.1%)	**2,198 (86.2%)**	<0.001
	True	981 (16.3%)	**347 (18.4%)**	281 (17.9%)	353 (13.8%)	
**Housing in notes**	False	1,798 (29.9%)	552 (29.2%)	**682 (43.5%)**	564 (22.1%)	<0.001
	True	4,213 (70.1%)	1,339 (70.8%)	887 (56.5%)	**1,987 (77.9%)**	

Only 3,515 patients (17.1%) in our population had billing codes indicating housing instability; this only represents 58.5% (n = 3,515) of patients with housing instability identified from any of our data sources. There were 65 patients who had “homeless” as the first address line, which was 6.6% of our address-based results. 8.2% of our population had a generic “Lexington, KY” or equivalent address that did not specify an address which does not necessarily imply a housing issue. 51.2% of patients with these generic addresses had housing issues identified by other methods. We detected 16.3% of those with unstable housing as having addresses directly corresponding to a community resource from our curated list.

**Figure 1.**
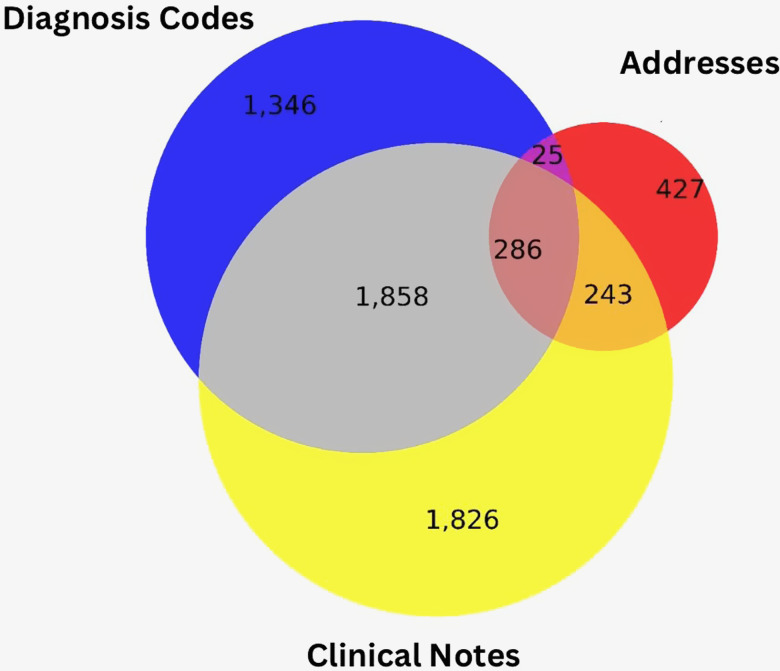
Unique patients (6,011 total) identified having housing issues by intersection of data source: diagnosis codes (3,515 total or 58.5%), addresses (981 total or 16.3%), and clinical notes (4,213 total or 70.1%).

**Figure 2.**
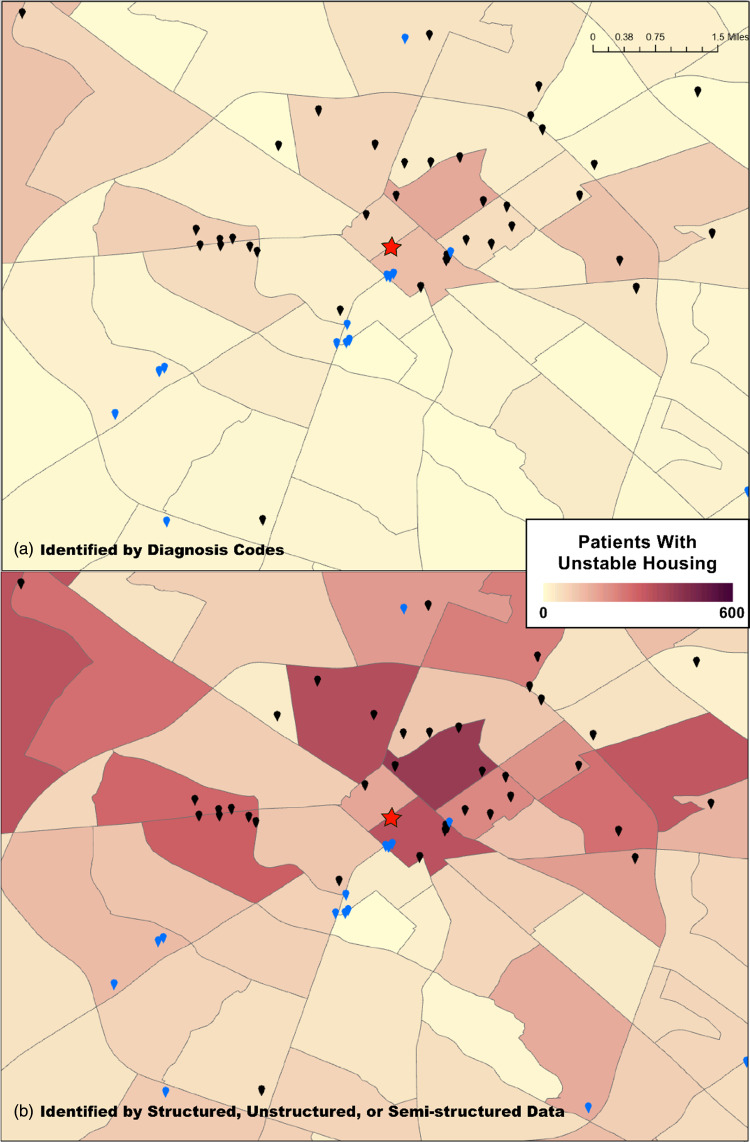
Patients with unstable housing in Fayette County, Kentucky, when (***a***) using only diagnosis codes or (***b***) when using diagnosis codes, clinical notes, or address data. Black pins are locations of housing-related community resources; blue pins are locations of hospitals, clinics, and emergency departments in our healthcare network; administrative boundaries are census tracts. The red star is the city center of downtown Lexington.

Six thousand and eleven patients in our study population (29.2%) had housing issues when merging signals from all three data sources. Fig. 1 demonstrates intersecting results of each method. 59.9% of patients with housing issues have documentation originating from a single source; only 286 (3.4%) had evidence in all three sources and less than a third had evidence from more than one source. This suggests that all three data sources are needed to understand housing issues; 41.5% of our population with housing issues are identifiable by analyzing addresses only (7.1%), clinical notes only (30.4%), or either one (4%). Fig. 2 visualizes how these differences are distributed within our community and shows the magnitude of underrepresentation for those with housing issues when only considering diagnosis codes. Similar results were observed within the stimulant-related group (17.5%–30.7%), the opioid group (8.1%–16.2%), and the group with both (34.8%–54%). As demonstrated in Fig. 2, there are implications for linking patient records to geographic units such as census tracts. If only diagnosis codes were used, 111 census tracts (7.8%) would be missed and considered absent of individuals with housing instability. Four hundred and seventy-eight census tracts (33.6%) saw increases in the number of individuals. Within these tracts, increases of up to 171 additional individuals were counted as having housing instability using results from address and note data; on average, 8.7 additional patients were added to each census tract.

Our validation review sample corresponded to 300 distinct individuals (4.9% of our population with housing instability). The reviewers unanimously agreed with the extractions in 239 out of 300 cases (79.7%), but the consensus varied by extraction method. NER and keyword methods had the highest total agreement (93% and 86%, respectively), but shelters were only 60% concordant; a Fleiss’ Kappa score of 0.35 suggested only fair agreement, which largely stemmed from ambiguity around what role a shelter or SUD treatment facility was serving in a patient’s life.

Table 3 describes how different methods of utilizing the clinical notes contributed to our housing instability totals from Table 2; keyword matching was the largest contributor. Approximately half of this population had addresses corresponding to known housing resources. Method was statistically significant (*p* < 0.001) for SUD type using Fisher’s exact tests.

**Table 3.** Evidence of housing instability in notes by method

		Overall	Stimulants	Opioids	Co-DX	*P*-value
**Total population**		4,213	1,339	887	1,987	
**Shelters by name**	False	2,084 (49.5%)	**742 (55.4%)**	439 (49.5%)	903 (45.4%)	<0.001
	True	2,129 (50.5%)	597 (44.6%)	448 (50.5%)	**1,084 (54.6%)**	
**Keywords**	False	610 (14.5%)	126 (9.4%)	**219 (24.7%)**	265 (13.3%)	<0.001
	True	3,603 (85.5%)	**1,213 (90.6%)**	668 (75.3%)	1,722 (86.7%)	
**Named-entity recognition**	False	3,996 (94.8%)	1,281 (95.7%)	**867 (97.7%)**	1,848 (93.0%)	<0.001
	True	217 (5.2%)	58 (4.3%)	20 (2.3%)	**139 (7.0%)**	

Table 4 describes demographics for those identified as having housing issues by data source. Sex and age were statistically significant (*p* < 0.001) for all data sources (diagnosis codes, addresses, and notes). Race was only significant (*p* < 0.001) for those with diagnosis codes and notes. Poisoning events related to stimulants or opioids (ICD10 T40.* and T43.6) were similarly identified in 17.1%–20% of the population across data sources; however, poisonings were only statistically significant for those with housing issues identified from diagnosis codes and notes (*p* < 0.001) using Fisher’s exact tests. Thousand and ninety-nine of our population’s 4,427 poisoning episodes (24.8%) were among those with evidence of housing instability; this represents 18.2% of our population with housing instability having had prior poisoning episodes. Only 703 of these 1,099 (63.9%) had a housing-related diagnosis code, which further highlights the importance of address and note data sources.

**Table 4.** Housing instability demographics by data source

		Overall	Diagnosis code		Address		Notes	
**Number of unique patients**		6,011	3,515		981		4,213	
**Sex**	Male	3,297 (55.3%)	2,041 (58.8%)	*p* < 0.001	**626 (64.3%)**	*p* < 0.001	2,258 (53.6%)	*p* < 0.001
	Female	2,666 (44.7%)	1,429 (41.2%)		348 (35.7%)		**1,955 (46.4%)**	
	Other or Unknown	1 (0.0%)	**1 (0.0%)**		0 (0%)		0 (0%)	
**Race**	White	5,207 (86.6%)	3,039 (86.5%)	*p* < 0.001	828 (84.4%)	*p* > 0.05	**3,661 (86.9%)**	*p* < 0.001
	Black	664 (11.0%)	378 (10.8%)		**129 (13.1%)**		495 (11.7%)	
	Other	10 (0.2%)	4 (0.1%)		**3 (0.3%)**		7 (0.2%)	
	Unknown	130 (2.2%)	**94 (2.7%)**		21 (2.1%)		50 (1.2%)	
**Age group (years)**	18–24	554 (9.3%)	245 (7.1%)	*p* < 0.001	**105 (10.8%)**	*p* < 0.001	429 (10.2%)	*p* < 0.001
	25–34	1,897 (31.8%)	967 (27.9%)		323 (33.2%)		**1,473 (35.0%)**	
	35–44	1,651 (27.7%)	**998 (28.8%)**		265 (27.2%)		1,172 (27.8%)	
	45–54	1,128 (18.9%)	**751 (21.6%)**		190 (19.5%)		735 (17.4%)	
	55-64	592 (9.9%)	**412 (11.9%)**		68 (7.0%)		345 (8.2%)	
	65+	142 (2.4%)	**98 (2.8%)**		23 (2.4%)		59 (1.4%)	
**Stimulant or opioid poisoning**	False	4,912 (81.7%)	2,812 (80.0%)	*p* < 0.001	802 (81.8%)	*p* > 0.05	**3,491 (82.9%)**	*p* < 0.001
	True	1099 (18.3%)	**703 (20.0%)**		179 (18.2%)		722 (17.1%)	

## Discussion

The nuances of housing stability are dynamic and complex by nature in healthcare data; our overarching goal was to demonstrate how multiple real-world data sources contribute to the identification of housing instability. Adding unstructured data nearly doubled the number of patients identified as having unstable housing.

The largest increase in housing status identification occurred from analyzing clinical notes. Both matching by keyword and by shelter names produced a substantial number of patients that were not detected otherwise. Our NER method underperformed, and all matches were available through other means; upon review, we learned that not all extracted named entities mapped to concepts in SNOMED CT due to the limitations of using a dictionary approach to concept matching. If the concept did not exist in SNOMED CT, the named entity would not produce a corresponding match, despite being detected. This creates bias in our NER results in that successful matches are limited to our target vocabulary, SNOMED CT, which may lack a comprehensive vocabulary for housing issues. We leave improving this model for future work, but we acknowledge that NER matches demonstrated higher accuracy and agreement between our manual adjudicators, which suggests that this method may give results with high precision at the cost of lowering recall.

We demonstrate that real-world local context is important in identifying those with disrupted living environments and that shelters providing temporary housing can be explicitly identified by name. We interpreted clinical notes that mention a shelter by name as an implication of the patient having a housing need. Many of these are in the form of “discharged to Shelter-X” as mentioned above, but there are indirect mentions that we also assumed implied a housing need. For example, “Social worker printed out information about two facilities, Shelter-X and Shelter-Y, and gave to patient;” this sentence is ambiguous on whether the patient stayed at either shelter, but it does imply that a provider perceived a housing need. Differences in demographics and risk of housing instability among SUD cohorts may be confounded by other factors. For example, more men than women have evidence of homelessness from address data (64.3% vs. 35.7%); the stimulant cohort has a higher percentage of men than others (59.7%) and has a higher number of individuals with address data implying housing issues. Men stay homeless longer than women on average [30] which increases their chance to stay at a shelter during our study period and more likely to use a shelter address for correspondence; additionally, our community has specific male-only shelters with recovery programs. We wish to further explore discrepancies in clinical documentation of housing instability across SUD cohorts as future work.

The most difficult cases when identifying by shelter names emerged when shelters served multiple roles, such as providing temporary housing and residential substance use treatment, which does not require homelessness as a condition for admittance; manual review did reveal some instances where shelters were used strictly for substance rehabilitation by the context given in the note. For this reason, searching for shelter names may overestimate housing instability unless residential treatment is included in the semantics of having a disrupted living environment. Address methods require curation of locations for community housing resources; for our study, this was a manual process using known data sources for our service area. Obtaining this information may be difficult for larger jurisdictions; however, if this information exists online, it could potentially be requested or scraped as part of a larger automation effort to improve the ease of implementation.

There are several vignettes from our study that are instructive. First, negations did impact the success of keyword matching and led to incorrect housing status assignment. For example, “asked if she was homeless and she denied,” but this phrase did not occur otherwise outside of the sample. Second, we found true false positives, such as “found a homeless person sleeping in her bathroom,” but these examples were uncommon and represented less than one percent of our sample and were so narrow in phrasing that they did not manifest otherwise. Third, our manual review found that language around housing is difficult and nonstandardized, which is far more problematic than false positives. For example, our team considers “has stable housing including homeless shelter” as paradoxical, as homeless shelters imply housing instability, and this provides data to advocate for better and more consistent clinical documentation. Fourth, we observed clinical documentation of “elective” homelessness, such as “patient was living on the streets but does have a home and multiple dogs,” this situation is relatively rare but highlights the complexity of housing circumstances. Fifth, the manual review demonstrated the difficulty of considering prior history of housing issues; one example documented an episode of homelessness that occurred several years ago.

All sources of evidence for housing issues have limitations. Diagnosis codes underrepresent the homeless population [13,15], which is confirmed in our study as 41.5% of individuals identified as homeless through other means lacked a diagnosis code; we did not validate the diagnostic accuracy of the ICD-10-CM diagnostic codes. Our address data were limited to the most recent address. Furthermore, our list of homeless resources was taken as a snapshot in time and may not reflect resources available during the entire 4-year study period. For these reasons, we were unable to examine the temporal relationship between the EHR address and housing issues. Our clinical notes are inherently limited to only what was documented within the note; we observed that 38.3% of patients having diagnosis codes for homelessness had no accompanying clinical documentation within the unstructured notes.

Many of the homeless shelters that serve our community’s housing needs also serve other roles, such as transitional living support or substance-related rehabilitation. This limitation does not impact our original goal of wanting to identify those with higher risk factors for SUDs and overdose; patients interacting with a shelter are already at a higher risk for substance-related issues regardless of *why* that interaction occurs because of the known association between homelessness, SUDs, and overdose [13]. Because of this association, we need comprehensive, real-world evidence of housing issues using multiple data sources. Our address and NER methods could be adopted by clinical data warehouse (CDW) teams to improve the identification of those with housing issues. In fact, our team is responsible for geocoding UKHC records on behalf of its CDW team and UK’s Center for Clinical and Translational Sciences; this data, in turn, is made available to others for enterprise reporting and research. We see an opportunity for a quality improvement project; our methods depend on reliable data, either accurate addresses or clinical documentation. Table 3 suggested that method of documentation is inconsistent across different SUD cohorts; Table 4 suggested that demographic characteristics of patients are related to how housing is documented. For example, it is not immediately clear why males have a higher proportion of housing documented as address data, but it suggests that care is needed when collecting address information during clinical administration to avoid bias. The importance of consistent documentation is further demonstrated by 18.3% of our population with housing issues having experienced a poisoning related to stimulants or opioids; clinical documentation potentially leads to better coordination of follow-up care and appropriate social services.

## Conclusion

The number of patients identified as having housing issues nearly doubled when including data sources for structured and semi-structured data; therefore, it is abundantly clear that translational use cases needing real-world evidence must consider diverse data sources. We advocate that real-world local context is paramount when processing unstructured data due to either the large occurrence of homeless shelters mentioned by name in clinical notes or the large number of patients with residential addresses corresponding to a shelter. Our study underscores the importance of analyzing multiple facets of text data from multiple data sources to get a comprehensive understanding of a patient’s SDOH.
